# Incremental high power radiofrequency ablation with multi-electrodes for small hepatocellular carcinoma: a prospective study

**DOI:** 10.1186/s12876-024-03358-w

**Published:** 2024-08-21

**Authors:** Sungjun Hwang, Jae Hyun Kim, Su Jong Yu, Jeong Min Lee

**Affiliations:** 1 Department of Radiology, Inje University Ilsan Paik Hospital, Goyang, Republic of Korea; 2 Department of Radiology, Seoul National University Hospital, 101, Daehak-ro, Jongno-gu, Seoul, Republic of Korea; 3 Department of Internal Medicine and Liver Research Institute, College of Medicine, Seoul National University, Seoul, Republic of Korea; 4 Institute of Radiation Medicine, Seoul National University Medical Research Center, Seoul, Republic of Korea; 5 Department of Radiology, Seoul National University College of Medicine, Seoul, Republic of Korea

**Keywords:** Tumor ablation, Multiple applicators, Image fusion, Hepatocellular carcinoma

## Abstract

**Abstract:**

Radiofrequency ablation (RFA) offers a minimally invasive treatment for small hepatocellular carcinoma (HCC), but it faces challenges such as high local recurrence rates. This prospective study, conducted from January 2020 to July 2022, evaluated a novel approach using a three-channel, dual radiofrequency (RF) generator with separable clustered electrodes to improve RFA’s efficacy and safety. The study employed a high-power, gradual, stepwise RFA method on HCCs (≤ 4 cm), utilizing real-time ultrasound-computed tomography (CT)/magnetic resonance imaging (MRI) fusion imaging. Involving 110 participants with 116 HCCs, the study reported no major complications. Local tumor progression (LTP) and intrahepatic remote recurrence (IRR) rates were low, with promising cumulative incidences at 1, 2, and 3 years for LTP (0.9%, 3.6%, 7.0%) and IRR (13.9%, 20.5%, 31.4%). Recurrence-free survival (RFS) rates were similarly encouraging: LTP (99.1%, 96.4%, 93.0%) and IRR (86.1%, 79.5%, 68.6%). This innovative gradual, incremental high-power RFA technique, featuring a dual switching monopolar mode and three electrodes, represents an effective and safer management option for small HCCs.

**Trial registration:**

*clinicaltrial.gov* identifier: NCT05397860, first registered on 26/05/2022.

## Introduction

Hepatocellular carcinoma (HCC) is a leading cause of cancer-related deaths worldwide [1]. Currently, image-guided radiofrequency ablation (RFA) or microwave ablation (MWA) is widely accepted as an effective curative treatment for very early-stage HCC (≤ 2 cm, single) or early-stage HCCs (< 3 cm, up to three in number) as suggested by guidelines from major international societies [2–4].

Several studies have demonstrated that the size of the ablative margin influences the development of local tumor progression (LTP) after ablation, with a typical target margin of 0.5–1.0 cm for liver tumor ablation [5–8]. Recent research suggests that achieving a larger ablative margin (> 1 cm) through microwave ablation (MWA) may significantly reduce the likelihood of LTP [9]. This advantage is attributed to MWA’s superior capabilities compared to RFA, including faster heating, higher temperature achievement, and reduced heat sink effect [9, 10]. However, most current RFA devices are limited by a single generator with a maximum power output of 200 to 250 watts (W), which may impede their ability to create an adequate safety margin. Increasing the power output beyond 300 W, combined with simultaneous energy delivery through three electrodes, could substantially improve the creation of a sufficient safety margin [7, 11].

**Fig. 1:**
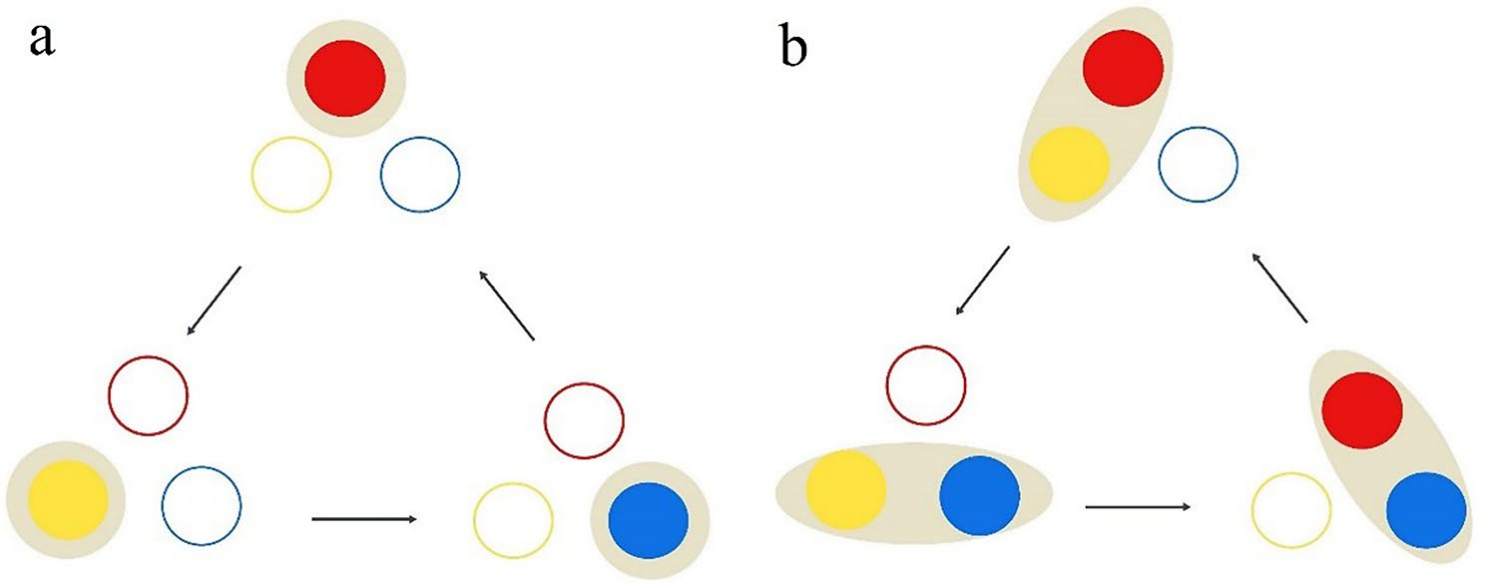
Single-switching monopolar mode and dual-switching monopolar mode. (**a**) In single-switching monopolar mode, radiofrequency energy is delivered one of three electrodes and is switched to adjacent electrode based on impedance increase. (**b**) In dual-switching monopolar mode, radiofrequency energy is delivered to one electrode of pair of electrodes at a time and switching mechanism is similar to that of single-switching monopolar mode

To enhance the efficiency of ablation procedures, a novel three-channel, dual-generator radiofrequency (RF) system has been developed [12]. This system is capable of generating up to 400 W of power, operating in dual switching monopolar (DSM) mode with a separable clustered electrode. The details of three-channel RFA with the separable clustered electrode and DSM mode have been described in previous studies [13–15]. In brief, the RF system is designed to counteract the impedance rise experienced during RFA with a single electrode, while also harnessing the synergistic benefits of three applicators to boost the efficacy of the ablation process through rapid heating [16] (Fig. 1).

Contrary to initial expectations that high-power DSM-RFA would reduce the incidence of local recurrence by efficiently creating a larger ablation zone [17], recent studies have shown that DSM-RFA failed to show superiority over single-switching monopolar (SSM) RFA (~ 200 W) in the treatment of HCC [12]. Highlighting the need for enhanced RF energy delivery efficiency and impedance control, there is also concern that high-power RF may elevate intratumoral pressure, risking malignant cell dispersal around the ablation zone [18].

We hypothesize that high-power RFA, employing a gradual increase in RF energy, will optimize energy delivery to HCC, enhancing both the efficacy and safety of the procedure by maintaining lower pressure and preventing rapid impedance rises.

Thus, this study aims to prospectively evaluate the rates of LTP and intrahepatic remote recurrence (IRR) for small (≤ 4 cm) HCCs treated with high-power RFA guided by real-time ultrasound-CT/MRI fusion imaging. The procedure utilizes a separable cluster electrode and gradual increment delivery in DSM mode.

## Materials and methods

### Compliance with ethical standards

This single-center, prospective study received approval from the Institutional Review Board of Seoul National University Hospital (IRB No. 1909-086-1064), and written informed consent was obtained from all participants who were enrolled in the study (*clinicaltrial.gov* identifier: NCT05397860, first registered on 26/05/2022). All study data are available for further scrutiny and can be obtained from the corresponding author upon request.

### Study design

The primary endpoint was the cumulative 3-year local tumor progression (LTP) rate after RFA. The secondary endpoint was the cumulative 3-year intrahepatic remote recurrence (IRR) rate after RFA. The tertiary endpoint was the cumulative recurrence-free survival (RFS) rates after RFA.

In this single-center, single-arm, prospective interventional study, we assessed participants with small nodular HCCs (≤ 4 cm) for potential enrollment in the study from January 2020 to July 2022. The inclusion criteria were as follows: (a) contrast-enhanced CT or MRI within 60 days prior to RFA; (b) presence of HCC, with a size ranging from 1 to 4 cm; (c) liver function categorized as Child–Pugh class A or B; (d) age between 20 and 85 years. The exclusion criteria for this study were: (a) presence of three or more malignant hepatic tumors; (b) largest tumor size exceeding 4 cm; (c) tumors with macrovascular invasion and/or distant metastasis; (d) platelet count lower than 50,000 mm^3^, or international normalized ratio greater than 1.5 (prothrombin time > 1.5 times normal); (e) liver function classified as Child–Pugh class C; (f) clinically confirmed non-HCC diagnoses; and (g) follow-up periods of less than 12 months. The diagnosis of HCCs was based on noninvasive imaging criteria according to the Korean Liver Cancer Association-National Cancer Center Korea guidelines [19]. The identification of the ablation zone was carried out using preprocedural multiphasic liver CT or gadoxetic acid-enhanced liver MRI.

### Equipment

A separable clustered electrode with three active tips (Octopus RF electrode; STARmed) and a multichannel RF generator (VIVA multi–RF Generator; STARmed) with a maximum power of 400 W (2 amps of 200 W) were used. In our switching RF system, the active electrode was switched when the impedance increased 50 Ω above baseline or when the ablation time passed 30 s [15]. For the RFA procedure, we configured the RF generator to operate in a gradual incremental mode. The electric current was initiated at 60 W and then systematically increased by increments of 10 W every minute. During energy delivery, chilled normal saline was circulated in the lumen of the electrode to keep the active tip temperature at 20–25 °C. The detailed algorithm of energy application was followed as per the manufacturer’s instructions.

### Ablation procedures

RFA procedures were conducted by a single operator with 26 years of experience in liver tumor RFA, with the assistance of a resident or a clinical fellow. Conscious sedation was achieved using intravenous injections of fentanyl (50–200 µg), midazolam (2–5 mg), and ketamine (1.5 mg/kg). For guidance, we employed real-time ultrasound-CT/MRI fusion imaging (RS 85, Samsung Medison) [20]. Electrode placement was determined by the operators using fusion images, with the distance between electrodes adjusted according to tumor size, maintained at 2–3 cm.

In this prospective study, we employed oncologically focused ablation techniques, primarily the “no-tumor-touch” method and RFA with feeding vessel ablation [21]. The “no-tumor-touch” RFA technique involved inserting three electrodes outside the tumors, which were then sequentially activated to create an ablation zone [22]. This approach avoided direct tumor violation, potentially reducing the incidence of LTP [23]. For RFA with feeding vessel ablation, we first identified the tumor’s feeding arteries using color Doppler or contrast-enhanced ultrasound (CEUS) with Sonazoid (Daiichi-Sankyo, Tokyo, Japan) microbubbles. When visible, we inserted an electrode near the artery and performed ablation. We then confirmed tumor blood flow disappearance using CEUS. If we detected residual flow, we repeated the ablation until no blood flow was observed. Finally, we inserted an electrode into the tumor for conventional RFA.

The choice between “no-tumor-touch” ablation and conventional tumor puncture ablation was based on factors like the availability of sufficient peritumoral liver tissue for electrode placement and the feasibility of safely inserting the required three electrodes for “no-tumor-touch” ablation [24]. When inserting three electrodes was not feasible, the tumor puncture method was used, with a preference for placing the electrodes in the tumor’s periphery. For index tumors located near subsegmental portal vein or hepatic arterial branches, the RFA with feeding vessel ablation technique [21] was applied. RF energy was administered simultaneously to two out of three electrodes using an automatic, gradual incremental technique, varying from 60 to 200 W per electrode (up to a maximum of 400 W) over 8–12 min, with the ablation time adjusted based on tumor size and the number of electrodes used.

During ablation, the formation of an echogenic complex was closely monitored in relation to the virtual tumor region of interest on real-time ultrasound-CT/MRI fusion imaging. RF energy was applied for 8–18 min using an impedance-switching algorithm until the ablative zone covered the entire tumor and safety margin [15, 25]. If the safety margin around a “virtual” target was deemed insufficient (≤ 5 mm), electrode repositioning was performed [26]. Vital signs were continuously monitored throughout the procedure. At the end of the RFA, tracts of each applicator were ablated by maintaining the active tips at 90 °C while retracting the electrodes to prevent bleeding and tumor seeding.

### Immediate technical success assessment

Immediately following the ablation procedure, contrast-enhanced CT scans were performed on all participants to assess the technical success of the ablation and identify any potential complications. Technical success was defined as the complete ablation of the target tumor with a sufficient safety margin (> 5 mm) around the tumor. The assessment used a scoring system based on contrast enhancement patterns and the presence and extent of a safety margin around the treated tumor on post-ablation CT scans. Score 4 (Ideal technical success) indicated no contrast enhancement within the treated lesion and a safety margin of > 5 mm around the tumor without contrast enhancement. Score 3 (Technical success – Borderline) revealed complete tumor necrosis and a safety margin of 2–5 mm without contrast enhancement. Score 2 (Technical success – Incomplete) revealed complete tumor necrosis, but a safety margin of < 2 mm without contrast enhancement. Score 1 (Technical failure) indicated residual contrast enhancement within the treated lesion.

### Complications

We also evaluated the development of post-ablation complications and the duration of hospital stays by reviewing medical records and imaging studies. Complications were graded according to the Clavien-Dindo classification [27], where complications of grade IIIa or higher were classified as major, and all others were considered minor.

Major complications were defined as events increasing the level of care or lengthening the hospital stay [7]. If a patient died within 30 days after the RFA, it was regarded as a procedure-related death [7]. Post-ablation syndrome, which consists of transient and self-limiting symptoms of low-grade fever and general malaise, was also reported, but was not regarded as a major complication [7].

### Technique efficacy, local control, and progression assessment

For each patient, the first follow-up involved a contrast-enhanced CT or MRI, performed one month after the procedure. Technical efficacy was defined as the complete ablation of the index tumor, confirmed by one-month follow-up imaging [7]. Patients who achieved complete ablation of the index tumor at the one-month follow-up were subsequently monitored with contrast-enhanced CT or MRI scans every three months.

The efficacy rate refers to the percentage of target tumors successfully ablated after the initial ablation [28]. LTP was defined as the appearance of tumor foci showing arterial enhancement and portal or delayed washout for HCCs at the edge of the ablation zone on contrast-enhanced cross-sectional imaging [7, 29].

Recurrence of HCC following ablation was categorized into three groups; (a) LTP, characterized by the emergence of tumor foci at the edge of the ablation zone after the initial successful treatment; (b) IRR, defined as the presence of HCC in the liver at a site not contiguous with the ablation zone; (c) Extrahepatic metastasis, referring to the spread of HCC to locations outside the liver [7].

### Statistical analysis

The cumulative LTP, IRR, and RFS rates at 1 year, 2 years, and 3 years were calculated using the Kaplan-Meier method. The time-to-LTP, at tumor-level data, was calculated as the length of time after RFA during the first LTP. If a patient died without LTP, time-to-LTP was censored at the date of death. Patients who underwent surgical resection or transplantation before LTP development were censored from LTP evaluation on the operation day. RFS, at patient-level data, was defined as the length of time after RFA to death or the first recurrence of the HCC on follow-up imaging. Recurrence was classified as LTP with or without intrasegmental spread, IRR, and extrahepatic spread [7]. Statistical analyses were performed using the Statistical Package for the Social Sciences (SPSS) (version 27, IBM, Armonk, NY, USA) and MedCalc software (MedCalc version 20.0.23; MedCalc Software, Mariakerke, Belgium), with *p* values < 0.05 considered to indicate statistical significance.

## Result

**Fig. 2:**
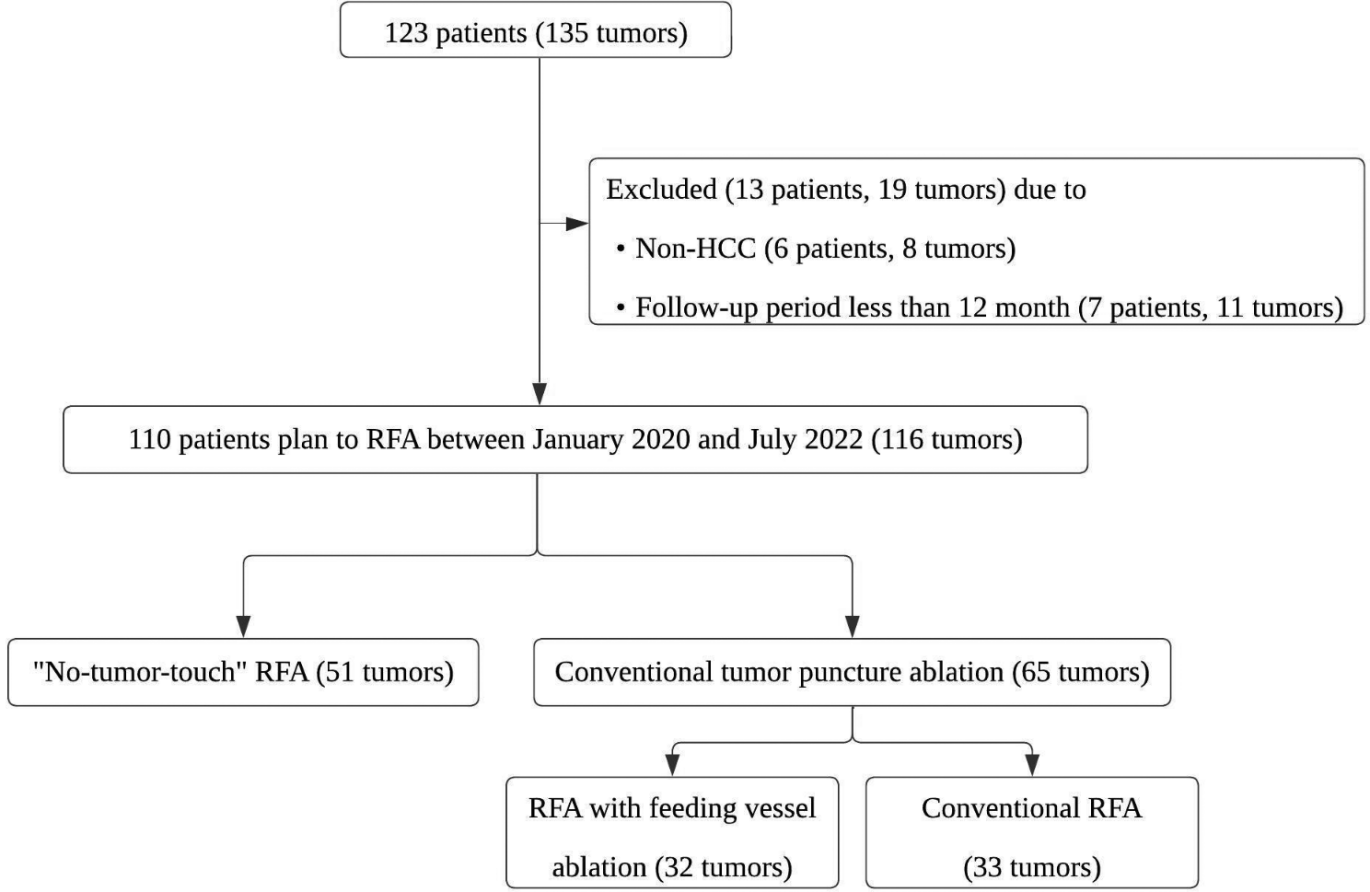
Flow diagram of included patients

Between January 2020 and July 2022, we identified 123 patients who met the inclusion criteria for our study. After excluding 6 patients with non-HCC diagnoses and 7 patients with follow-up periods of less than 12 months, our final study population consisted of 110 patients (Fig. 2). Among these 110 enrolled patients, six had two HCCs, resulting in a total of 116 tumors being treated with ablation. The baseline characteristics of all participants are summarized in Table 1.

**Table 1:** Participants characteristics

Characteristics	Data
Total patients (Tumor numbers)	110 (116)
Age (years)	66.4 ± 7.6
Sex	
No. of men	83 (75.5%)
No. of women	27 (24.5%)
Underlying liver disease	
Hepatitis B virus	81 (73.6%)
Hepatitis C virus	10 (9.1%)
Alcohol	19 (17.3%)
Child-Pugh class	
A	105 (95.5%)
B	5 (4.5%)
Alpha-fetoprotein level (ng/mL)	49.4 ± 228.3
Tumor location	
Right anterior section	48 (41.4%)
Right posterior section	45 (38.8%)
Left medial section	8 (6.9%)
Left lateral section	15 (12.9%)

*Note*^a^ is the Spearman-Brown coefficient, and ^b^ is the Cronbach alpha coefficient

### Technical success and efficacy of ablation

**Table 2** presents a comparative analysis of 110 patients with 116 tumors. The mean tumor size was similar across all techniques, showing no significant difference (*p* = 0.123). However, the “no-tumor-touch” technique resulted in significantly larger ablation diameters (53.1 ± 9.68 mm) compared to the other methods (*p* = 0.047). Although the ablation time tended to be longer with the “no-tumor-touch” technique, this difference was not statistically significant (*p* = 0.088). The distribution of 5 mm safety margin scores was similar across all techniques (*p* = 0.933).

**Table 2:** Comparative analysis of treated 110 patients with 116 tumors

Treated tumors (*n* = 116)	“No tumor touch” (*n* = 51)	RFA with feeding vessel ablation (*n* = 32)	Conventional RFA (*n* = 33)	*p*
Mean tumor size (cm)	1.9 ± 0.76	1.5 ± 0.49	1.6 ± 0.47	0.123
Ablation diameter (mm)	53.1 ± 9.68	47.6 ± 8.74	48.8 ± 8.72	**0.047**
Ablation time (min)	9.8 ± 4.49	7.7 ± 3.51	8.4 ± 3.20	0.088
**5 mm score**				0.933
Score 4 (Complete)	35 (68.6%)	22 (68.8%)	24 (72.7%)	
Score 3 (Borderline)	13 (25.5%)	9 (28.1%)	7 (21.2%)	
Score 2 (Incomplete)	3 (5.9%)	1 (3.1%)	2 (6.1%)	
**Recurrence of tumor**				**< 0.001**
No recurrence	45 (88.2%)	22 (68.8%)	15 (45.5%)	
Local tumor progression	1 (2.0%)	1 (3.1%)	2 (6.1%)	
Intrahepatic remote recurrence	5 (9.8%)	9 (28.1%)	16 (48.5%)	
**Treatment after recurrence**				0.363
Radiofrequency ablation	5 (14.3%)	6 (17.1%)	9 (25.7%)	
Transarterial chemoembolization	1 (2.9%)	5 (14.3%)	9 (25.7%)	
Note.—*p* values less than 0.05 indicate statistical significance.				

Notably, there was a significant difference in tumor recurrence patterns among the techniques (*p* < 0.001), with the “no-tumor-touch” technique showing the lowest recurrence rate (11.8%: 2.0% LTP and 9.8% IRR). Conventional RFA was associated with the highest rates of recurrence rate (54.5%: 6.1% LTP and 48.4% IRR).

All participants attained complete ablation of the index tumor as evaluated at the 1-month follow-up CT or MRI (efficacy, 100% [110/110]). During a median follow-up of 41.0 months (range, 35.4–46.6 months), LTP occurred in 4 tumors and IRR occurred in 30 tumors. The recurrent tumors were treated using transarterial chemoembolization (*n* = 15) and RFA (*n* = 20). There was no significant difference in the choice of treatment after recurrence (*p* = 0.363).

### Post-ablation complications

**Fig. 3:**
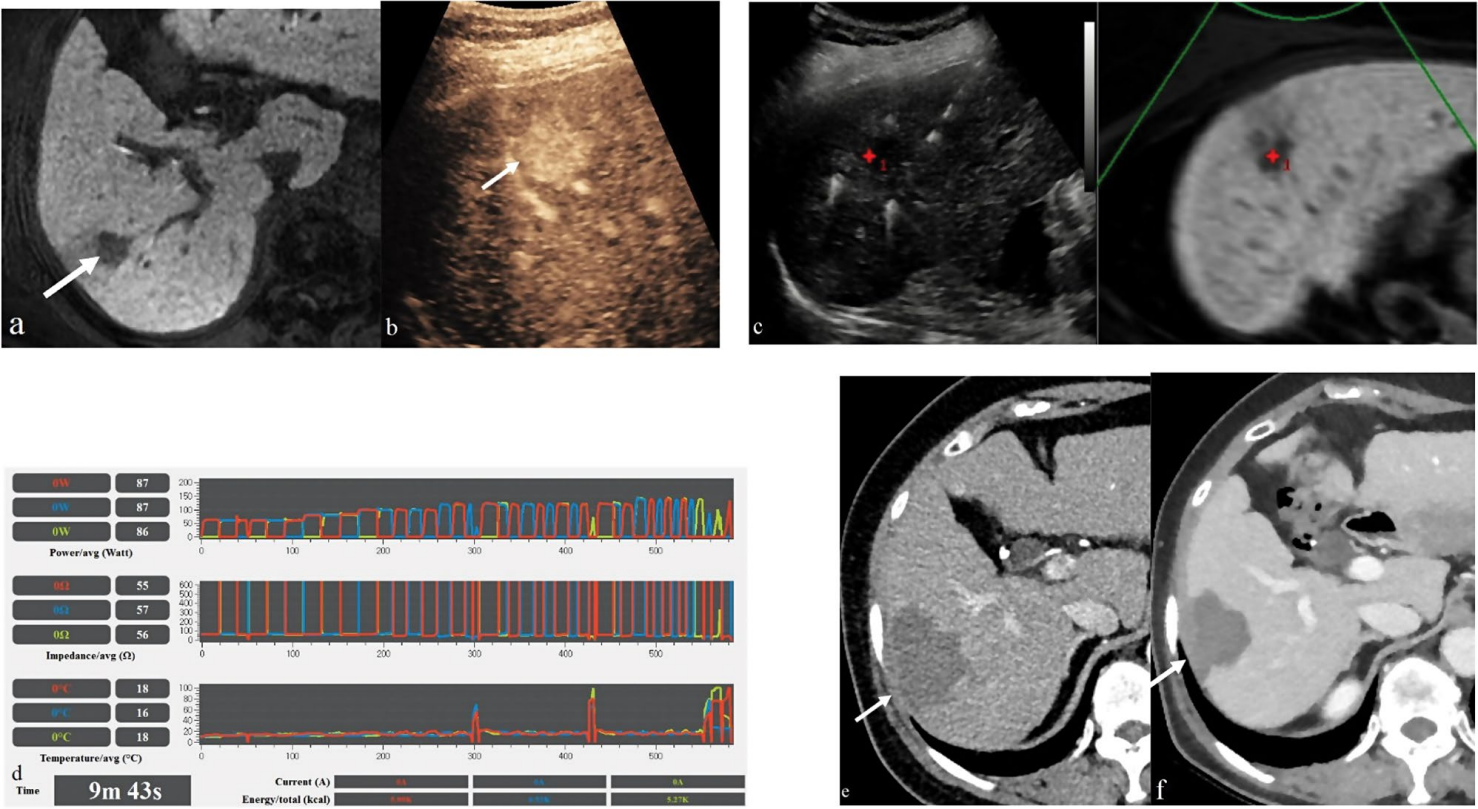
“No-tumor-touch” radiofrequency ablation in a 74-year-old woman with a 1.6-cm hepatocellular carcinoma (HCC) and hepatitis B-related cirrhosis. (**a**) Hepatobiliary phase image of gadoxetic acid-enhanced MRI shows a 1.6-cm low signal intensity HCC (arrow) in segment VI of the liver. (**b**) Contrast-enhanced ultrasound (Sonazoid) images well visualize the tumor (arrow). (**c**) Real-time ultrasound-computed tomography (CT)/magnetic resonance imaging (MRI) fusion imaging shows the low echoic target tumor. (**d**) In the panel, the active electrode was switched when the ablation time reached approximately 20–30 s. The electric current was initially set at 60 W and then systematically increased in 10 W increments every minute. The average impedance of each electrode was measured at 55–57 Ω, and this was correlated with the active electrode switch occurring when the impedance increased by 50 Ω above the baseline. The total ablation time was 9 min and 43 s, with the mean total energy of each electrode measured at 5.93 Kcal. (**e**) Portal venous phase coronal image of immediate CT scan shows complete ablation of the target tumor and tumor-bearing portal vein branches with a sufficient safety margin (> 5 mm). (**f**) No local tumor progression was observed at 39-month follow-up CT

The “no-tumor-touch” ablation technique, which refers to ablation without puncturing the tumor with an electrode (Fig. 3), was performed. Complete ablation of the index tumor was achieved in 99.1% of patients (*n* = 109) on immediate CT scans. The only exception was one patient who required repeated ablation due to severe diaphragmatic adhesion resulting from a previous surgical procedure. However, this patient subsequently obtained a sufficient margination of > 5 mm (Score 4) after prompt repeated ablation.

Out of the 110 patients, six experienced minor complications (5.5%, 6/110): grade I (low-grade fever and general malaise, *n* = 4) and grade II (transient diaphragmatic injury, *n* = 1; peripheral portal vein branch injury near the tumor, *n* = 1). One patient had a major complication: grade IIIb acute cholecystitis (*n* = 1). The average hospital stay was 1.2 ± 1.1 days (range, 1–9 days).

**Fig. 4:**
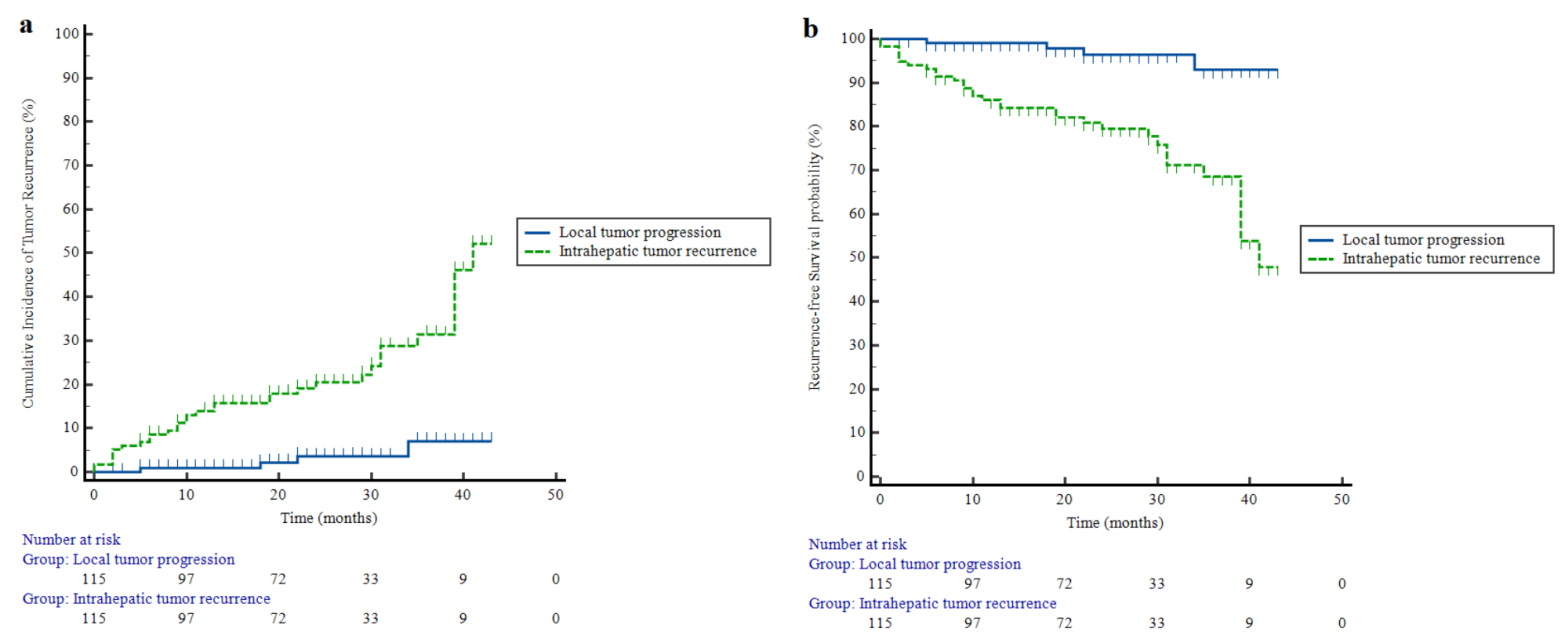
Kaplan-Meier estimation of the cumulative incidence of (**a**) tumor recurrence and (**b**) recurrence-free survival after incremental high power radiofrequency ablation in a hepatocellular carcinoma ≤ 4.0 cm

### Cumulative incidences of LTP, IRR, and RFS

The estimated cumulative incidences of LTP at 1 year, 2 years, and 3 years were 0.9%, 3.6%, and 7.0%, respectively. For IRR, the incidences were 13.9%, 20.5%, and 31.4% (Fig. 4). There were significant differences between the three groups in recurrence rates (*p* < 0.001). Additionally, there were no cases of extrahepatic metastasis during the follow-up period. The estimated RFS rates for LTP at 1 year, 2 years, and 3 years were 99.1%, 96.4%, and 93.0%, respectively. For IRR, the rates were 86.1%, 79.5%, and 68.6%, respectively.

## Discussion

This prospective study evaluated the effectiveness and safety of gradual, incremental high-power RFA using a separable clustered electrode and a three-channel generator for treating small HCCs (≤ 4 cm). Our results showed that the stepwise delivery of RF energy up to 400 W was feasible and achieved a 100% efficacy rate at the one-month follow-up. With a median follow-up of 41.0 months, the estimated cumulative incidences of LTP at 1, 2, and 3 years were 0.9%, 3.6%, and 7.0%, respectively. These results appear promising when compared to historical data on conventional RFA (10–25%) and are comparable to reported outcomes for MWA (5–10%) [30–34]. However, direct comparisons should be made cautiously due to differences in study design and patient populations.

Several factors may potentially contribute to the observed LTP rates with high-power RFA. The enhanced energy delivery (up to 400 W) through DSM mode may help manage impedance rise, potentially reducing residual tumor post-ablation [17]. The gradual energy increment, applied through peripherally placed electrodes, could facilitate early blockade of blood supply and maintain lower intratumoral pressure [24], possibly preventing the dispersion of malignant cells [35]. The use of a separable clustered electrode and real-time ultrasound-CT/MRI fusion imaging allowed accurate electrode placement with ideal interelectrode distance [36], while three electrodes may have optimized energy distribution and ablation shape.

We employed oncologically driven techniques such as the “no-tumor-touch” method [24, 33] in 51 patients and RFA with feeding vessel ablation [37] in 32 patients. The “no-tumor-touch” method, preferred for tumors with ample peritumoral parenchyma and clear access, may offer enhanced local tumor control [23, 24, 33] due to sufficient safety margins, improved thermal efficiency, and prevention of track seeding [24]. In our study, the “no-tumor-touch” group had significantly larger ablation diameters (*p* < 0.05) and lower incidence of LTP and IRR compared to the conventional RFA group, suggesting a potential positive impact on patient outcomes.

According to historical control data from randomized prospective comparative studies at our institution, the “no-tumor-touch” RFA method has shown promising results in reducing LTP rates compared to conventional RFA. Suh YS et al. [33] reported that the 1-year and 3-year cumulative LTP rates for conventional RFA were 11.8% and 21.3%, respectively. In contrast, the “no-tumor-touch” RFA group demonstrated significantly lower cumulative LTP rates of 5.6% at both 1 and 3 years. Similarly, Park SJ et al. [34] found that the 1- and 2-year estimated cumulative incidences of LTP were 3.5% and 3.5% in the “no-tumor-touch” RFA group, compared to 8.9% and 13.5% in the conventional RFA group. These findings align with our results, suggesting that the “no-tumor-touch” method may provide superior local control compared to conventional RFA. Our study further supports this hypothesis, demonstrating the potential benefits of this technique in reducing LTP rates.

Limitations of this single-center, single-arm study include potential bias and the lack of a control group for direct comparison. Multi-center studies with larger sample sizes, control groups, and long-term follow-ups are necessary to validate these results, assess sustainability, and evaluate the long-term impact on patient survival.

In conclusion, the gradual incremental high-power RFA technique using a separable clustered electrode and a three-channel, dual generator system shows promise as an effective and safe approach for treating small HCCs. However, further comparative studies with diverse patient populations and long-term follow-ups are essential to substantiate these findings and assess the long-term benefits of this advanced ablation technique relative to conventional methods.

## Data Availability

The datasets used and/or analyzed during the current study are available from the corresponding author on reasonable request.

## Abbreviations

HCC: Hepatocellular carcinoma
RFA: Radiofrequency ablation
MWA: Microwave ablation
CT: Computed tomography
MRI: Magnetic resonance imaging
LTP: Local tumor progression
IRR: Intrahepatic remote recurrence
RFS: Recurrence-free survival
SSM: Single-switching monopolar
DSM: Dual-switching monopolar
